# Human-animal interaction as a social determinant of health: descriptive findings from the health and retirement study

**DOI:** 10.1186/s12889-018-5188-0

**Published:** 2018-03-09

**Authors:** Megan K. Mueller, Nancy R. Gee, Regina M. Bures

**Affiliations:** 1 Department of Clinical Sciences, Cummings School of Veterinary Medicine at Tufts University; Tufts Institute for Human-Animal Interaction; Jonathan M. Tisch College of Civic Life at Tufts University, North Grafton, MA USA; 2 Department of Psychology, State University of New York, Fredonia, NY USA; 3 WALTHAM Centre for Pet Nutrition, Leicestershire, UK; 4 Eunice Kennedy Shriver National Institute of Child Health and Human Development, National Institutes of Health, Bethesda, MD USA

**Keywords:** Human-animal interaction, Companion animals, Aging

## Abstract

**Background:**

We focused on human-animal interaction (HAI) as an important aspect of social functioning at the individual level, framing this emerging field from a public health perspective.

**Methods:**

Using data from the Health and Retirement Study (HRS) 2012 HAI module, we describe the characteristics of pet ownership in a population of older adults, and examine the relation between pet ownership and multiple mental and physical health indicators such as health status, depression, and physical activity.

**Results:**

Of the 1657 participants in our subsample, approximately half (51.5%) reported being pet owners; the majority owned dogs or cats, and most had only one pet. Pet ownership was significantly associated with a higher likelihood of ever having had depression, with pet owners being 1.89 times more likely to have experienced depression. However, pet ownership was not associated with having experienced depression within the last week.

**Conclusions:**

The findings from this study could indicate a relationship between pet ownership and depression, but it is impossible to determine the directionality of that relationship. It is possible that owning a pet may put a person at an increased risk of developing depression, or individuals who are at risk, or who have already developed depression, may acquire a pet as a way of managing their depressive symptoms. The findings of this study provide an initial step in contributing to our understanding of the relationship between companion animals and the social, physical, and mental well-being of the HRS study population. Future research should include measures of HAI in longitudinal, population-based surveys.

## Background

Public health traditionally has focused on animals in terms of environmental health, reflecting concerns that they may serve as vectors of disease. Yet research has offered little evidence of pets as significant contributors to human disease [1–3]; rather, it has been suggested that pet ownership may be beneficial in promoting health outcomes [4, 5]. While Beck and Meyers [6] concluded that research on the benefits of animal companionship to public health was needed, little has been done in the United States to move this work to the population level.

To provide a public health perspective on the impact of companion animals in the lives of older adults and to characterize the relationship between pet ownership status and its potential relation to human health, we reframe the study of Human-Animal Interaction (HAI) in the context of social determinants of health. These broadly include neighborhood, education, and socioeconomic status, all of which may be mediated by protective factors, including social support. We also focus on a specific dimension of HAI, companion animal (pet) ownership. Pets may facilitate the formation of social connections [7] and the development of social capital [8], and many pet owners report attachment to their pets [7].

In this paper we focus on a population-based sample of Americans aged 50 and older. The human population is rapidly aging and expected to nearly double globally by the year 2050; the number of older people is expected to exceed the number of children for the first time in 2047 [9]. Most people over age 65 in the United States live independently [10], and estimates indicate that 14% of them have companion animals [11]. As individuals age, the benefits and importance of social support for the maintenance of both physical and emotional health may increase. Pet ownership may provide emotional health benefits for persons over age 65 [12] and could thus potentially facilitate successful aging.

### HAI and healthy aging

Quality of life is a key element in successful aging, a term synonymous with productive and healthy aging [12]. A recent meta-analysis of research on successful aging identified four key components: 1) avoiding disability and disease, 2) having high functioning mental/cognitive/physical capacity, 3) being actively engaged, and 4) being able to psychologically adapt later in life [13]. This work suggests that social and psychological dimensions may be as important as physical and cognitive function. As a social determinant of health, human-animal interaction may be a potential element of successful aging.

A growing body of research in HAI suggests that interacting with a companion animal can offer a range of potential benefits to older adults. Interacting with a companion animal (particularly a dog) can reduce depression [14–17] or elevate mood [18], decrease anxiety [16, 19], lower blood pressure [20], and increase social interaction [21–24]. In general, researchers have less information on the impact of owning a companion animal compared to interacting with one within a therapeutic setting (such as animal-assisted therapy), due to the complex nature of these relationships and how they evolve over time. One fundamental challenge of such research is that drawing causal inferences about the impact of pet ownership on various aspects of psychological and physical health can require randomly assigning pet ownership to people. However, there are ethical and logistical concerns with this approach, and generally people prefer to determine for themselves whether or not to own a pet, and if opting for ownership, they prefer to select their own pet species.

To date, only a single study has randomly assigned pet ownership: Allen, Shykoff, and Izzo [5] conducted a clinical trial in which 48 hypertensive individuals were randomly assigned to either an experimental condition (an ACE inhibitor -Lisinopril, and pet ownership) or a control condition (only the ACE inhibitor). The ACE inhibitor was effective at reducing resting blood pressure, but failed to reduce cardiovascular responses to stress, whereas pet ownership was effective at reducing cardiovascular reactivity to mental stress. This study provides convincing evidence of the potential for pet ownership to play a causal role in mediating physiological responses to psychological stressors. In fact, in 2013 the American Heart Association summarized the results of many studies investigating the relation between pet ownership and risk of cardiovascular disease (CVD), concluding that “[p]et ownership, particularly dog ownership, may have some causal role in reducing CVD risk (Level of Evidence: B)” ([25], p. 4).

The majority of research on this topic is correlational, describing associations between pet ownership and physical and psychological health outcomes. Recent literature reviews on HAI and human aging suggest that pet ownership is positively associated with several measures of psychological and physical health in order adults (e.g., [26]), but the evidence base is small and results are often contradictory. For example, in two survival studies following myocardial infarction, one found that pet owners were more likely to die than their non-pet-owning counterparts [27]; the other found that pet-owners were less likely to die than the non-pet-owners [28].

A similarly conflicted pattern has emerged in mental health studies. McConnell and colleagues [29] reported that pet owners demonstrated higher levels of emotional well-being, self-esteem, and social inclusion, but Peacock and colleagues [30] link pet ownership to higher levels of psychopathology. Herzog [31] describes a number of potential reasons for such conflicted findings, including lack of rigorous research designs, self-report biases, the tendency to ignore non-significant findings, as well as a variety of issues related to comparing pet-owners with non-pet owners. A fundamental question that cannot be teased apart in the absence of random assignment of pet ownership status is whether obtaining a pet makes people healthier or if healthier people opt to get a pet.

Given that assigning pet ownership status is challenging in the best of scientific circumstances, our next best approach is to better understand the similarities and differences between people who self-select to be pet owners or not. Large, nationally representative studies provide this opportunity. Therefore, the purpose of this study was to explore patterns of pet ownership in a population-representative survey of older adults, and to explore the relationship between pet ownership and indicators of health and well-being.

## Methods

### Data

We used data from the 2012 wave the Health and Retirement Study (HRS),^1^ an ongoing biennial longitudinal cohort study of approximately 20,000 Americans aged 50 and older and, if married, their spouses, regardless of the spouse’s age. HRS respondents are re-interviewed at two-year intervals and the sample has been replenished multiple times since the study originated in 1992. The HRS contains detailed information on family structure and composition, health, and labor force participation. The HRS 2012 also included an experimental module on HAI. Participants are randomly assigned to one experimental module per wave; of the 20,554 respondents in this wave of data collection, 2037 were assigned to the HAI module. The HRS 2012 was the first nationally representative survey in the United States to include measures of HAI in this detail. Questions from the module were not repeated in 2014, limiting our analyses to the cross-section.

Our sample includes 1657 of the participants who responded to the HAI module and were also 50 years of age or older (younger participants were excluded from this analysis). The HRS module samples are random subsamples of the full HRS sample, therefore we used the 2012 HRS respondent weight in our analyses. Sample weights are calculated using the probability of selection for the individual and a post-stratification factor adjusting for non-response (based on age, gender, race/ethnicity, geographic differences), as well as a non-participation adjustment for each specific wave. Module non-response among participants who completed the interview is low, which means that while this weight does not account for non-response to the modules themselves (only to the 2012 interview), we anticipate that our estimates will be unbiased.

### Pet ownership, attachment, and health outcome measures

#### Pet ownership

To assess the characteristics of pet ownership in older adults, several items from the HAI module were used. Participants were asked to report whether they currently owned a pet, types of pets (dog, cat, small mammal, bird, fish, reptile, or other), number of each type of pet, and number of years they have had pets.

#### Pet attachment

Attachment to pets was measured using the Pet Attachment Questionnaire [32], originally developed for use in older adults. This measure includes six items: “Do you consider your pet a friend?” “Do you talk to your pet?” “Would you say that owning a pet adds to your happiness?” “Do you talk to others about your pet?” “Do you often play with your pet?” and “Does your pet know how you feel about things?” Each item had a dichotomous response option of yes (coded as 2), or no (coded as 1); per the original coding from Garrity and colleagues [12], item scores were summed to create an attachment score with a range of 6 to 12. In this sample, the scale demonstrated good reliability (Cronbach’s α = .72).

#### Health outcomes

Overall health was measured using a five point self-report rating from 1 = Excellent to 5 = Poor. Participants were also asked if they had ever experienced depression (yes/no) and if they had experienced depression within the last week (yes/no). Physical activity was measured using three variables, assessing frequency of mild, moderate, and vigorous physical activity; response options ranged from “every day” to “hardly ever or never.”

#### Demographics

In addition to pet ownership and health outcomes, we also utilized several of the demographic items in the survey; e.g., participants reported their marital status (married, separated, divorced, widowed, never married, or other) and their housing situation: owning (or buying) a home, renting a home, living rent-free with relatives, or other.

### Analyses

Analyses were conducted using SPSS software. Descriptive analyses (mean, median, standard deviation, frequencies) were used to characterize HAI in aging and to demonstrate the importance of pet ownership as a context of interest in the aging population. For summary statistics, the weighted results are presented unless otherwise indicated, accompanied by the unweighted sample size (N). Weighted regression models were used to test differences between pet owners and non-pet owners on age, gender, and health status. Age was stratified into three groups; 50–69, 70–84, and 85 and older. Complex samples logistic regression models were used to test differences between pet owners and non-pet owners on depression and home ownership. For all comparisons, values of *p* < .05 were considered significant.

## Results

### Characteristics of pet ownership in older adults

Of the 1657 participants in our subsample, the majority were female (58.6%), and age ranged from 50 to 101 years (*M =* 67.29, *SD =* 10.44); 935 participants were 50–69 years old (67.6% of the weighted sample), 625 were 70–84 years old (27.6% of the weighted sample), and 97 were 85 years or older (4.8% of the weighted sample). Table 1 presents percentages of the weighted sample, separated by gender, within the three age groups. Across the sample, there were no significant gender differences in rates of pet ownership (*F* [1,1606] = 0.25, *p =* 0.62). However, age category was a significant predictor of pet ownership (*F* [2,1605] = 25.53, *p <* 0.001), with adults ages 50–69 (OR = 4.97; 95% CI [2.88, 8.58]) and 70–84 (OR = 2.53; 95% CI [1.46, 4.39]) significantly more likely to own a pet than those 85 years or older. As indicated, most males across age groups tended to be married and own their own homes, with no significant differences across age groups in marital status (*F* [2623] = 1.93, *p =* 0.15) or home ownership (*F* [2623] = 2.50, *p =* 0.08). For females, age was a significant predictor of being married (*F* [2980] = 23.54, *p <* 0*.*001), with 50 to 69 year olds being 9.03 times as likely (95% CI [4.56, 17.91]) and 70 to 84 year olds being 4.73 times as likely (95% CI [2.37, 9.44]) to be married compared to the 85 and older age group. Similarly, age was a significant predictor of owning a home for females (*F* [2980] = 5.07, *p* = 0*.*006), with 50 to 69 year olds being 2.55 times as likely (95% CI [1.43, 4.56]) and 70 to 84 year olds being 2.29 times as likely (95% CI [1.27, 4.13]) to own a house compared to the 85 and older age group.

**Table 1:** Health and demographics, by age and gender (weighted)

	50–69 years old		70–84 years old		85 + years old	
	Male	Female	Male	Female	Male	Female
Pet Owners (%)	57.2%	58.3%	37.3%	43.9%	19.0%	22.5%
Married (%)	66.8%^1^	61.9%^2^	75.0%^1^	45.9%^2^	62.0%^1^	15.2%^2^
Own Home (%)	79.8%^3^	79.2%^4^	85.4%^3^	77.0%^4^	65.0%^3^	59.6%^4^
Health Status (*M, SD*)	2.56 (1.00)	2.57 (1.06)	2.84 (1.06)	2.84 (1.05)	3.17 (1.10)	2.98 (0.99)
Ever Depressed (%)	17.3%	32.1%	9.7%	20.6%	3.5%	11.9%
Depressed in last week (%)	13.1%	13.2%	4.2%	13.0%	2.8%	9.4%
Mild Physical Activity (% every day or once a week)	75.7%	83.6%	67.4%	76.2%	54.9%	73.6%
Moderate Physical Activity (% every day or once a week)	69.1%	65.0%	60.7%	56.6%	61.7%	24.4%
Vigorous Physical Activity (% every day or once a week)	50.9%	36.1%	38.8%	28.5%	4.1%	20.8%

^1^Comparison within males; *ns, p =* 0.15

^2^Comparison within females; *p < .*001, 50 to 69 and 70 to 84 age groups significantly more likely to be married than 85 and older age group

^3^Comparison within males; *ns, p =* 0.08

^4^Comparison within females; *p =* 0.006, 50 to 69 and 70 to 84 age groups significantly more likely to be married than 85 and older age group

In total, 51.5% of respondents reported being pet owners; the majority of these owned dogs (68.4%) and/or cats (45.6%), in addition to the 5.3% who owned birds, 6.1% owned fish, 1.0% owned small mammals, and 0.7% had reptiles. Most participants (75.2%) reported having only one animal, although total number of pets ranged from 1 to 50 (*M =* 2.98, *SD =* 5.19). Many participants were long-term pet owners, with 33.8% reporting having pets for 10 or more years. See Table 2 for pet ownership characteristics by age group. Pet owners were also significantly more likely to own a home (*N* = 583; 75.8%), as compared to non-pet owners (*N* = 562; 61.0%), *F* (1, 1400) = 9.98, *p* = 0.002.

**Table 2:** Descriptive characteristics (weighted) of pet ownership in older adult pet owners (*n* = 757), by age

	Number of Pets *M* (SD)	Dog owners (%)	Cat owners (%)	Attachment *M* (SD)
50–69 years old	3.09 (5.46)	69.7%	44.8%	11.35 (1.20)
70–84 years old	2.79 (4.36)	65.4%	49.4%	11.55 (0.88)
85 + years old	1.15 (0.36)	51.7%	36.1%	11.02 (1.85)
Total sample	2.98 (5.19)	35.2%	23.5%	11.39 (1.15)

Table 3 presents weighted percentages of pet owners, separated by gender and age grouping. Within this group of pet owners, the percentage of males owning dogs did not differ across the three age groups (*F* [2275] = 0.25, *p =* 0.78), nor did the percentage of females (*F* [2457] = 1.56, *p =* 0.21). The percentages for cat ownership are similar across age categories for females (*F* [2457] = 0.03, *p =* 0.97), but there is a significant effect of age on cat ownership in males (*F* [2276] = 7307.89, *p <* 0*.*001), with cat ownership dropping to zero in the oldest age category for males. When comparing both genders and age categories, percentage of bird ownership is highest among males in the 85+ age group.

**Table 3:** Type of pet ownership in older adult pet owners, by age and gender (weighted)

	50–69 years old		70–84 years old		85 + years old	
	Male (%)	Female (%)	Male (%)	Female (%)	Male (%)	Female (%)
All Pet Owners	41.9%	58.1%	37.9%	62.1%	22.3%	77.7%
Dogs	71.8%^1^	68.2%^2^	67.2%^1^	64.2%^2^	75.9%^1^	44.8%^2^
Cats	38.1%^3^	49.6%^4^	48.1%^3^	50.2%^4^	0.0%^3^	46.5%^4^
Birds	7.6%	3.7%	7.1%	3.8%	24.1%	0.0%
Fish	10.2%	4.7%	2.2%	3.4%	0.0%	8.8%
Small Mammals	1.6%	1.0%	0.0%	0.4%	0.0%	0.0%
Reptiles	0.3%	1.2%	0.0%	0.3%	0.0%	0.0%

^1^Comparison within males; *ns, p =* 0.78

^2^Comparison within females; *ns, p =* 0.21

^3^Comparison within males; *p < .*001

^4^Comparison within females; *ns, p =* 0.97

Older adult pet owners indicated engaging positively with their companion animals in several ways. They reported being highly attached to their pets; 88.3% considered their pet a friend, 96.1% reported talking to their pet on a regular basis, 92.8% felt that their pet adds to their overall happiness, 88.1% regularly talk to others about their pet, 83.6% reported playing with their pet, and 81.4% thought their pet knows how they feel. Overall, attachment was high (*M =* 11.39, *Mdn =* 12.00, *SD* = 1.15, range 6 to 12). In addition, of dog owners, 63.3% regularly walk their dogs (*M* = 1.61 times per day, *SD* = 1.48).

### Pet ownership and health outcomes

Regression analyses indicated no significant differences between pet owners and non-pet owners on general health status, B = 0.01, *t*(1605) = 0.12, *p =* 0.90. Pet ownership was significantly associated with the likelihood of ever having had depression *F*(1, 1596) = 16.75, *p* < 0.001, with pet owners 1.89 times more likely to have experienced depression than non-pet owners 95% CI [1.39, 2.57]. However, there were no differences between pet owners and non-pet owners on recent depression (having experienced depression within the last week), *F*(1, 1605) = 2.33, *p =* 0.13.

## Discussion

The results of this descriptive analysis of the HRS 2012 data provide us with a unique opportunity to better understand pet ownership in older adults and to consider possible links to social determinants of health as well as physical and psychological health status. These links merit further investigation and will likely have an increasingly important impact on public health as our population ages.

Analyses of the HRS 2012 HAI module reveal that not only is pet ownership common in older adulthood (51.5% of respondents, and 43.2% of respondents 65+), it appears to be much more common than previously estimated^2^ (14% among those 65+). The HRS 2012 sample included individuals 50 years or older, so one could hypothesize that pet ownership drops off as people get older, thus explaining the difference in the two percentages. In fact, Table 1 reveals that the weighted percentage of pet owners in the HRS 2012 sample does decrease across the three age categories to the lowest point in the 85+ age group. However, the percentage of pet owners in the 85+ category is still higher than the 14% estimated previously. Further, pet owners in the HRS 2012 sample reported near-ceiling levels of attachment to their pets. Over 80% of all pet owners reported that they consider their pet a friend, talk to their pet regularly, feel their pet adds to their happiness, talk to others about their pet and play with their pet. These results are similar to previous research documenting the relation between pet attachment support and loneliness for older women [33]. Combined, these results indicate that pet ownership among older adults is not only widespread, it appears to be emotionally engaging and important to their social functioning.

It is not surprising that dogs and cats were the most frequently reported pets owned by older adults; this is consistent with numbers reported in the general population [2]. This sample of older pet owners also tended to be long-term pet owners, suggesting a lifelong choice to include animals in their lives, although most reported currently owning only one pet. The pet owners in this sample tended to be younger than the non-pet owners. The drop-off in pet ownership observed in the 85+ category may represent a time in life when older adults find it increasingly difficult to care for a pet or move into an assisted living or nursing home facility that does not allow pets; regulations vary greatly from state to state [34], and it is not uncommon for senior housing facilities to have policies that prohibit pet ownership. The pet owners in the HRS 2012 sample were also more likely to own their own homes than the non-pet owners in the sample. This finding could be similarly explained by the age-related decline in pet ownership such that older adults (who are more likely to be living in assisted-living facilities) are less likely to own their own home and pets are often not allowed in rental or assisted-living properties. Another potential explanation is that pet owners are more affluent than non-pet owners and therefore more likely to own a home. This is consistent with previous research showing the same trend in the general population.

It has been suggested that pet ownership may be associated with maintaining one’s health into older adulthood; in this sample, more than 60% of dog owners regularly walked their dogs, which may be indicative of maintaining a physically active, healthy lifestyle. However, there was no significant difference between pet owners and non-owners on overall health status. In fact, in this sample, pet ownership was a significant predictor of the likelihood of ever having experienced depression. This could indicate a relationship between pet ownership and depression, but it is impossible to determine the directionality of that relationship. Do people become depressed because they have opted to include pets in their lives or do people who are depressed opt to acquire a pet as a way of treating their depression? In the HRS sample, current depression (in the last week) was not associated with pet ownership status. In other words, previous, but not current, depression is related to pet ownership status. One potential explanation is that the ongoing companionship of the pet may function to alleviate depressive symptoms; on the other hand, the loss of a previous pet and the corresponding loss of companionship could exacerbate depressive symptoms.

## Conclusions

Given that more than half of the participants in our subsample of the HRS 2012 HAI module are pet owners, and the data to date on health benefits in the elderly and other populations, it seems clear that the health implications for older adults should be more fully explored. We advocate a public health perspective on HAI research in older adults. Pet ownership may serve as a social determinant of health by increasing the potential for social interaction and social support.

In addition to the potential benefits of pet ownership for older adults, there are also potential health challenges unique to older persons that should be explored in future research. For example, dog and cat ownership can be associated with increased risk for falls [35]. Future work should address the costs and benefits of pet ownership for older adults. There may be strategies other than pet ownership, such as interacting with the pet of a family member or friend, that provide benefits of HAI with minimal associated costs. Intervention studies that identify effective strategies for optimizing mutually-beneficial human-animal relationships in aging adults as well as across the life course are needed.

Overall, the cross-sectional nature of these data limits our ability to draw any causal conclusions about the relationship between HAI and health outcomes. To overcome these limitations, researchers should consider adding HAI questions, such as those used in the HRS module, in future population and public health surveys collecting data on respondents across the life course over repeated waves of data collection. Such longitudinal data would allow for complex multivariable, multi-level models to assess potential confounders and effect modifiers impacting the relationship between pet ownership and health outcomes. Pet ownership questions as well as questions about exposure and interaction with animals as well as the relationships between individuals, pets and social interaction will contribute to a clearer understanding of the impact of HAI on social interaction and social support. Only by consistently measuring the impact of HAI at the population level will we begin to fully understand the public health implications of pet ownership and other forms of HAI.

## Abbreviations

HAI: Human-animal Interaction
HRS: Health and Retirement Study 2012
CVD: Cardiovascular disease
ACE: Angiotensin-converting-enzyme (as in ACE inhibitors)
NIA: National Institute on Aging

## Footnotes

1 The HRS is supported by the National Institute on Aging (NIA U01AG009740) and the Social Security Administration
